# Harnessing Conformational Dynamics and Computational Design to Generate Novel Enzymes

**DOI:** 10.1063/4.0000840

**Published:** 2025-10-27

**Authors:** Lynn Kamerlin

**Affiliations:** Georgia Institute of Technology, Atlanta, GA, USA

## Abstract

Understanding how new enzyme functions evolve, either on existing scaffolds, or completely *de novo* on previously non-catalytic scaffolds, is of great interest both from a fundamental biochemistry perspective, and from a biotechnological perspective. Several hypotheses have been put forward to rationalize enzyme evolution, one of which is that their conformational dynamics plays an important role in facilitating the emergence of new enzyme functions.^1, 2^ Such conformational dynamics, in turn, is an important feature that can be exploited in artificial enzyme design.^3-5^ In this talk, I will illustrate how we have engineered conformational dynamics to generate a *de novo* active site capable of catalyzing a non-natural reaction,^6^ with *k*_cat_/*K*_M_ ∼ 5 × 10^3^ M^−1^ s^−1^ and *k*_cat_ ∼ 10 s^−1^. Subsequent computationally-focused ultra-low-throughput screening using FuncLib^7^ allowed us to obtain a Kemp eliminase with a catalytic efficiency (*k*_cat_/*K*_M_ ∼2 × 10^4^ M^−1^ s^−1^ and *k*_cat_ ∼10^2^ s^−1^) approaching that of naturally occurring enzymes.^8^ Finally, more recently, we have extended our design strategy to include computational stability design guided by NMR and simulation,^9^ and our latest Kemp eliminase from this strategy has catalytic efficiency (*k*_cat_/*K*_M_ 4.3 × 10^5^ M^−1^ s^−1^ and *k*_cat_ ∼1700 s^−1^) exceeding the best Kemp eliminase designed to date using a proton abstraction mechanism,^10^ upon screening a small focused selection of variants. The broader implications of this for protein engineering will also be discussed.
